# Mandibular Posterior Anatomic Limit for Distalization in Patients With Various Patterns of Third Molar Impactions: A Three-Dimensional Cone Beam CT (CBCT) Study

**DOI:** 10.7759/cureus.50165

**Published:** 2023-12-08

**Authors:** Preethi Rajamanickam, Shantha K Sundari

**Affiliations:** 1 Orthodontics and Dentofacial Orthopedics, Saveetha Dental College and Hospitals, Saveetha Institute of Medical and Technical Sciences, Saveetha University, Chennai, IND

**Keywords:** regional acceleratory phenomenon, lingual cortex, posterior limit, distalization, molar impaction

## Abstract

Aim: The aim was to compare and evaluate the variation in the mandible’s posterior anatomic limits (MPAL) stratified by different third molar impaction patterns utilizing cone-beam CT (CBCT) in individuals with skeletal Class III malocclusion.

Methodology: The sample consisted of CBCT records of 80 samples of Class III patients categorized based on the pattern of their third molar impaction. The shortest linear distances from the distal root of the second mandibular molar to the inner cortex of the mandibular body were measured at the crown level, at the cementoenamel junction (CEJ), and at the depths of 4, 6, and 8mm from the CEJ, all parallel to the posterior occlusal line. The MPAL of the four groups were compared. The Shapiro-Wilk test for normality was performed. The Kruskal-Wallis test was performed to compare the MPAL distances between the groups.

Results: Significant differences were noted between the four groups at every level. MPAL distances decreased towards apex in all the groups and was least at the 8mm root level. The greatest MPAL distances were noted in the no-impaction group followed by the horizontal. At 8mm, the MPAL were 4.2+/-1.3 in Group 3 (Control), 3.3+/-0.9 in Group 1 and 2.7+/-0.05 mm in Group 2. Though the amount of space available for distalization was greatest for the mesioangular group at the crown level, it was least at the 8mm level compared to other groups.

Conclusion: MPAL distances were shortest at the root level in Class III patients who had mesioangularly impacted third molars and care should be taken before attempting distalization in them.

## Introduction

Temporary anchorage devices (TADs) have made it possible for dentition to move as intended and have helped clinicians recognize the clinical significance of distalization in nonextraction treatment. Utilization of the infrazygomatic area in the maxillary arch and buccal shelf area in the mandibular arch for mini-implant insertion has been gaining popularity owing to the sufficient bone density in this region [1,2].

TADs offer orthodontists the capability to achieve greater tooth movement compared to conventional methods [3,4]. Nonetheless, it's important to note that there are anatomical constraints limiting the extent of this movement. Traditionally, it was believed that the anterior border of the mandibular ramus in the lower arch and the maxillary tuberosity in the upper arch represented the posterior limits for distalizing the respective dentitions [5-7]. However, recent research employing three-dimensional (3D) cone-beam CT (CBCT) has revealed that the lingual cortex of the mandibular body acts as the posterior anatomical limit for distalizing the mandibular second molar. Also, distalization in either of the arches usually is preceded by extraction of third molars, be they completely erupted or impacted [8]. The removal of third molars is to facilitate tooth distalization by utilizing the socket space and also to occasionally make use of the regional acceleratory phenomenon [9,10,11]. Impacted mandibular third molars are the most commonly encountered teeth, with prevalence rates ranging from 16.7% to 68.6% [8,12-15] without any sexual predilection. Depending upon the angulation of the third molars impacted, they can be classified as vertical, mesioangular, distoangular, or horizontal [16]. However, the relation between the angulation of the third molars and the amount of distalization possible after their extraction is unknown. Though the removal of mandibular third molars frequently precedes mandibular distalization, there have also been cases where very little or no distalization occurred despite the removal of the third molars. Therefore, the aim of this research was to assess whether the specific impaction pattern of third molars has an impact on the available space for distalizing mandibular dentition.

## Materials and methods

This prospective study was conducted at the Saveetha University of Medical and Technical Sciences (SIMATS), Chennai, India, between the period of December 2021 to August 2022. Scientific Review Board approval number SRB/SDC/ORTHO-2003/22/121 was assigned to the study. The study consisted of pre-treatment CBCT records of 80 Class III patients categorized into four broad groups based on the pattern of third molar impaction and it was ensured that the following criteria were met: 1) adult skeletal Class III patients with a normodivergent facial profile (between 18 to 35 years of age); 2) no noticeable facial asymmetry; 3) with no evident alveolar bone loss. 4) No previous history of orthodontic treatment. CBCT records with grossly decayed or missing third molars were excluded. Radiographic assessment for bone loss involved calculating the distance between the cementoenamel junction (CEJ) to the alveolar crest (AC) which should be less than or equal to 1mm. The absence of crater-like or angular defects and increased periodontal ligament space were confirmed. Furthermore, CBCT records that showed changes in the contour of the alveolar crest, such as flattening or irregularity, were excluded. Sample size calculation was performed in G*Power software, Version 3.0, (Heinrich Heine University Düsseldorf, Düsseldorf, Germany). The sample size was derived using data from a study by Kim et al. on normodivergent class I individuals to establish the posterior mandibular anatomical limit for distalization [3]. The sample size was calculated to be 80 with a power of 90% and the level of significance was set up to be 0.05. Simple randomization was done to obtain 80 records from a pool of 120 records.

Group 1 comprised horizontal impaction (N=20). Group 2 comprised vertical impaction (N=20). Group 3 comprised mesioangular impaction (N=20). Group 4 comprised non-impaction (N=20).

Location of mandibular posterior limit 

**Figure 1:**
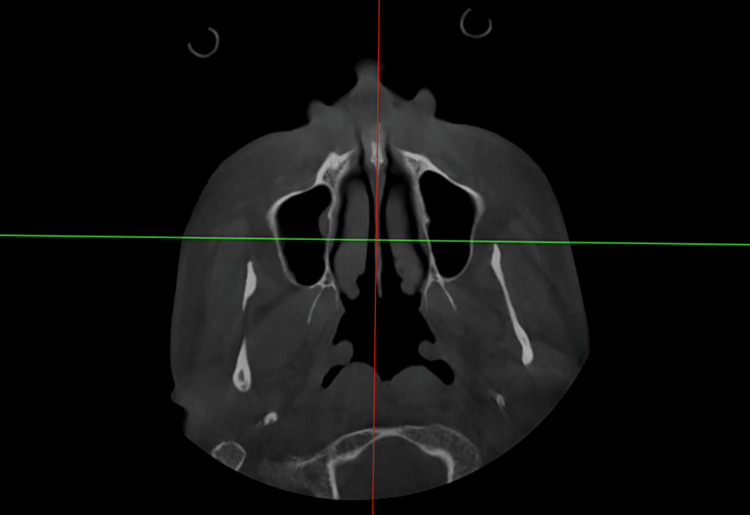
Midsagittal plane passing through the skeletal landmark crista galli in the CBCT CBCT: Cone Beam CT

CBCT records were analyzed using the Carestream 9600 software (Carestream Dental LLC, Atlanta, Georgia, United States) with the following settings; slices were 3.0 mm thick, 1.5 mm in pitch, 180 mA, and 120 kV. The CT images in Digital Imaging and Communications in Medicine (DICOM) format were converted to 3D images in Dolphin 3D imaging software (Patterson Dental Supply, Saint Paul, Minnesota, United States). The MPAL distance was assessed by measuring the span from the distal root of the second molar to the mandibular bone's lingual cortex as described by Choi et al. [17]. Orientation of the third image was done by aligning the midsagittal and mandibular planes. By penetrating through the crista galli, opisthion, anterior nasal spine, and the middle of the midsagittal plane, the mandibular incisor tip, was reoriented. The buccal cusp tip of the mandibular first molar and the lower incisor’s tip on the opposing side were both traversed by the mandibular plane (Figure 1).

**Figure 2:**
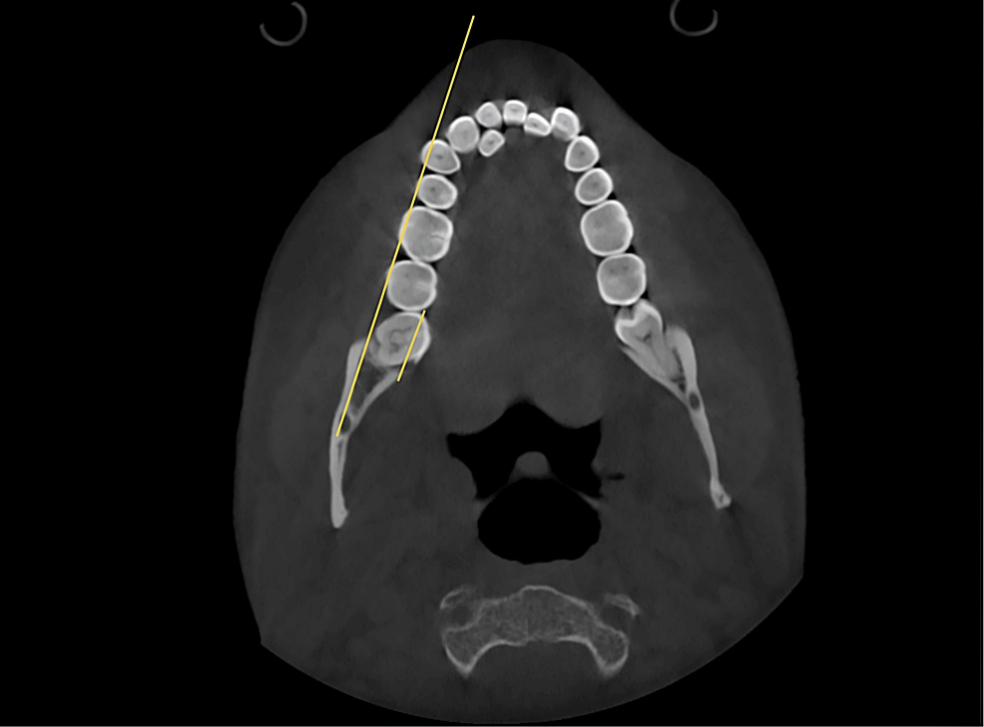
Posterior occlusal line passing through the buccal cusp tips of first and second molars. The short line represents the extent of MPAL, which is measured from the most distal point on the distolingual cusp, determined on the line passing parallel to the posterior occlusal line. MPAL: Mandibular posterior anatomic limit

**Figure 3:**
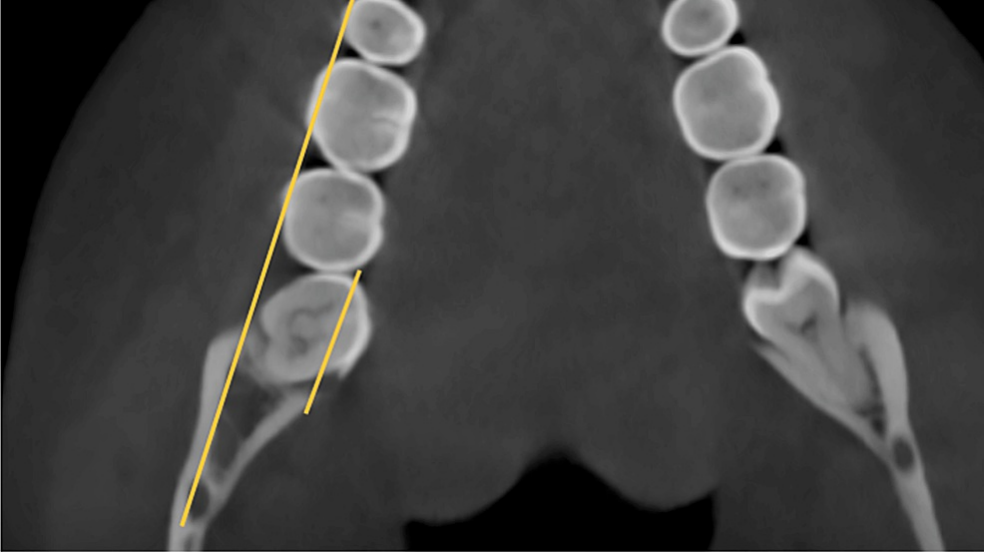
Zoomed-in view of the extent of MPAL MPAL: Mandibular posterior anatomic limit

**Figure 4:**
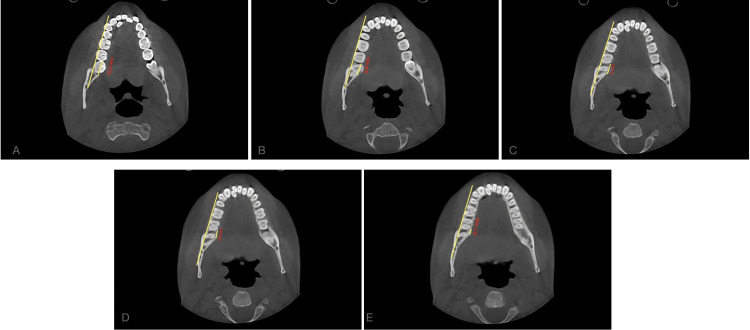
Control (Group 4); measurements taken at the (A) crown level, (B) at the CEJ, and at depths of (C) 4 mm, (D) 6 mm, and (E) 8 mm from the CEJ, all parallel to the posterior occlusal line CEJ - Cementoenamel junction

**Figure 5:**
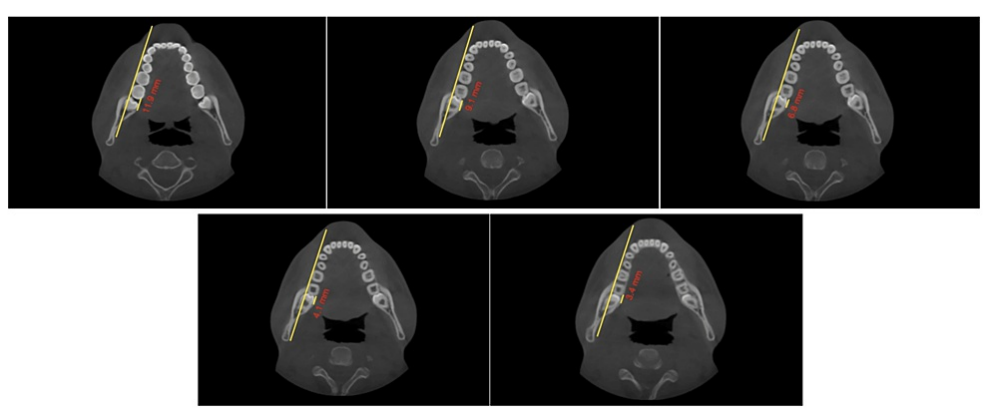
Group 1; measurements taken at the (A) crown level, (B) at the CEJ, and at depths of (C) 4mm, (D) 6mm, and (E) 8 mm from the CEJ, all parallel to the posterior occlusal line CEJ: Cementoenamel junction

**Figure 6:**
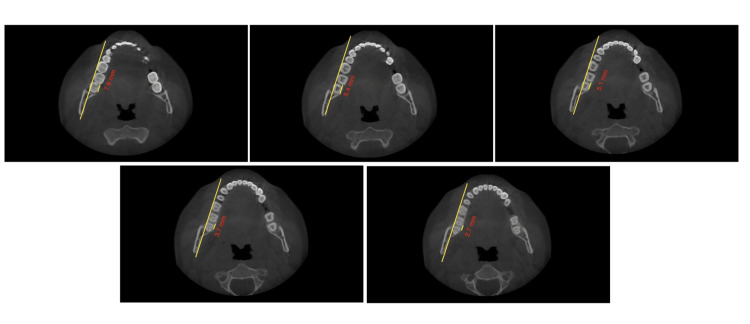
Group 2; measurements taken at the (A) crown level, (B) at the CEJ, and at depths of (C) 4mm, (D) 6mm, and (E) 8 mm from the CEJ, all parallel to the posterior occlusal line CEJ: Cementoenamel junction

**Figure 7:**
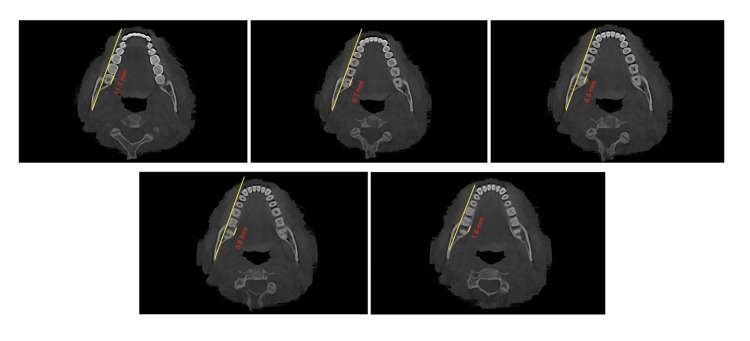
Group 3; measurements taken at the (A) crown level, (B) at the CEJ, and at depths of (C) 4mm, (D) 6mm, and (E) 8 mm from the CEJ, all parallel to the posterior occlusal line CEJ: Cementoenamel junction

The study involved measuring the shortest dimension of linear distances from the distal root of the second mandibular molar to the inner cortex of the mandibular body. These measurements were taken at the crown level, at the cementoenamel junction (CEJ), and at depths of 4mm, 6mm, and 8 mm from the CEJ, all parallel to the posterior occlusal line. Subsequently, the MPAL values between the four groups were compared. Two clinicians conducted the measurements independently, and the final value used was the average of their measurements (Figures 2, 3, 4, 5, 6, 7).

Statistical analysis 

For statistical analysis, the researchers utilized IBM SPSS Statistics for Windows, Version 23, (Released 2015; IBM Corp., Armonk, New York, United States). The sample size was determined using G*Power 3.0 software, with an estimated size of 80, based on an effect size of 0.1, a significance level of 0.05, and a power of 90%. The normality of the data was assessed using the Shapiro-Wilk test, and for parameter comparison, the Kruskal-Wallis test was conducted.

## Results

The Shapiro-Wilk test revealed that the data was non-parametric (p-value <0.05) and hence the Kruskal-Wallis test was performed to compare the MPAL distances at different levels for all four groups. The results of the current study concluded that significant differences were noted in the measurements between the four groups at every level. MPAL distances decreased as it moved apically below the CEJ in all the groups and was least at the 8mm root level. Three examiners independently performed the measurements and the average of their individual measurements was calculated and used for the study. Intra-examiner and inter-examiner reliability were high with an intra-class correlation coefficient of 0.99 and 0.97, respectively. The greatest MPAL distances were noted in the no-impaction group followed by the horizontal impaction group. When measured at the crown level, the MPAL were 9.1+/-4 in Group 1, 7.3+/-0.3 in Group 2, 12.5+/-0.9 mm in Group 3, and 10.8+/-3.3 in Group 4. At the CEJ level, MPAL were 7.2+/-2.1 in Group 1, 4.5+/-1 in Group 2, 9+/-0.8 mm in Group 3, and 9.3+/-2.6 in Group 4. At 8 mm, the MPAL were 3.3+/-0.9 in Group 1, 2.7+/-0.05 in Group 2, 1+/-0.7 in Group 3, and 4.2+/-1.3 in Group 4 (Control) (Table 1).

**Table 1:** Test results of Kruskal-Wallis test performed to compare the extent of mandibular posterior anatomical limit in all the four groups at the crown level, at the CEJ, at 4mm, at 6mm, and at 8mm apical to the CEJ *p-value significant CEJ: Cementoenamel junction

Parameter	Horizontal (Mean+/-SD)	Vertical (Mean+/-SD)	Mesioangular (Mean+/-SD)	Control (Mean+/-SD)	p-value
At crown level	9.1+/-4	7.3+/-0.3	12.5+/-0.9	10.8+/-3.3	0.01*
CEJ	7.2+/-2.1	4.5+/-1	9+/-0.8	9.3+/-2.6	0.00*
4mm	5+/-2	5.2+/-0.1	8.3+/-1.8	6.5+/-1	0.00*
6mm	3.5+/-1.4	3.5+/-0.4	3.2+/-0.6	5.4+/-1.2	0.00*
8mm	3.3+/-0.9	2.7+/-0.05	1+/-0.7	4.2+/-1.3	0.00*

## Discussion

The introduction of mini implants has proved to be a valuable treatment approach for molar distalization or en masse distalization with minimal patient compliance. Since posterior limits for molar distalization exist, the amount of distalization that can be achieved in any case differs between cases. Several factors have been found to account for the variation in the amount of possible distalization. Factors such as growth pattern, sagittal relationship between the mandible and maxilla were known to influence the anatomic limit for distaliation posteriorly.

In one of the studies, it was concluded that distinct skeletal patterns can result in distinct anatomical morphologies of the submandibular fossa and mylohyoid ridge, which together determine the MPAL [18]. Kim et al. (2021) and Hui et al. (2022) have presented findings regarding the mandibular posterior anatomic limit for distalization. Their research concluded that the anatomical characteristics of the mylohyoid ridge and submandibular fossa, which define the MPAL, can exhibit variations among different skeletal patterns [19,20]. Notably, their studies revealed that the mandibular posterior anatomic limit for distalization is shortest in individuals with hyperdivergent profiles. It's worth noting that a significant limitation in many previous studies lies in their exclusive focus on patients with skeletal Class I malocclusion and a normodivergent pattern. However, it's crucial to recognize that the demand for mandibular distalization is particularly high among Class III patients. While distalization is sometimes employed to address crowding in specific cases of Class I malocclusion, its primary application is in patients with skeletal Class III malocclusion.

Thus this study was carried out in Class III patients with average growth pattern in whom the mandibular third molars have fully erupted or partially/completely impacted. Four groups were involved in the study based on the pattern of impaction, but it is inevitable to note that patient records with distoangular impaction were not included in the study as there were not enough samples in this group to conduct the study. There were only two cases of distoangular impaction out of the 120 case records initially pooled for the study. This lack of a distoangular pattern of impaction was supported by the study by Pillai et al. which concluded that the distoangular pattern of third molar impaction accounts for only 5.7% of the overall third molar impaction rate [21].

The anatomic constraints of tooth movement must be considered during the treatment planning process. The orthodontic wall, well known as the alveolar bone housing, serves as a cortical bone plate in the alveolar bone structure. It covers the tooth and plays a crucial role in stabilizing the tooth during orthodontic treatment, preventing unwanted movement. Traditionally, it was believed that various regions, including the anterior palate, symphysis, maxillary tuberosity, and lingual cortex of the mandibular body, influence the extent of tooth movement [3]. Orthodontic tooth movement is impeded when the tooth encounters this cortical plate, and if the tooth continues to shift beyond this point, it can weaken the surrounding periodontal tissues, leading to a less favorable long-term prognosis for the tooth's function [22,23]. To address these challenges, particularly when tooth contact with the inner cortical plate becomes a concern, our study focused on evaluating the inner surface of the lingual cortex in the mandibular body. 

The amount of distalization that is feasible is calculated at the region of the root apex as MPAL is least at the root apex. This structural anatomy of the mandible accounts for this anatomic constriction in the bone housing at the apex [3,12,24]. The mylohyoid ridge, which marks the posterior borders, interferes with the mandibular second molar intrusion [18]. Because they act as a mandibular lower anatomic barrier in the posterior region, the mylohyoid ridge and submandibular fossa require specific consideration from orthodontists.

The results of the current study reveal that at the crown level, the amount of available space for distalization was maximum in those mandibles in which the third molars were mesioangularly impacted and least in those mandibles in which the third molars were vertically impacted. At 8mm above the CEJ, the MPAL were highest in those mandibles in which the third molars were fully erupted, followed by the horizontally impacted group, and were least in those mandibles in which the third molars were mesioangularly impacted towards the apex region (8mm below the CEJ). On the other hand, the MPAL distance at the crown level was highest for the mesioangularly impacted group, which progressively decreased to the least MPAL at 8mm as it descended. This variability could be attributed to the theory that the mesioangularly inclined third molar, tipping the crown of the second molar mesially and the root distally, creating an MPAL greatest at the crown level and least at the root level. So in general, the amount of space available for distalization was highest in those mandibles without third molar impaction followed by those mandibles in which the third molars were horizontally impacted, at the root level (8mm apical to the CEJ).

To summarize, at 8mm below the apex, the MPAL were 3.3+/-0.9 in Group 1, 2.7+/-0.05 in Group 2, 1+/-0.7 in Group 3, and 4.2+/-1.3 in Group 4 (Control). These results were in accordance with those of the MPAL distances derived in the previous studies, wherein Jing et al. reported an average available space for distalization of 4.85+/-1.78 and Hui et al. reported an average of 2.80 ± 1.96 mm at 3mm below the root level [3,17,20,25]. MPAL measurements of 2.7-2.8 mm at the level of 6mm below (apical) the root furcation were made by Choi et al. and Kim et al. reported distance at 8mm apical to the CEJ in this study, or 3.16+/-2.41mm [3,17,20]. These values obtained from our study appear to be appropriate and reliable as one of the previous studies observed in those patients with a Class III malocclusion revealed that the amount of the molar distalization varied between 3.2mm and 4.9mm [26]. There were individual variations in every case, hence CBCT exams might be required for precisely planning the distalization therapy for patients with Class III type of malocclusion. When the posterior accessible space for distalization on lateral cephalograms is less than 3.9mm, Kim et al. advised performing CBCT [3,27].

According to Pell and Gregory classifications from PG-A (the cusp tip of the third molar is at the same level as or higher than the occlusion plane of the second molar) to PG-C (the cusp tip of the third molar is below the neck of the second molar), PG-I (adequate space exists between the anterior border of the ascending ramus and the distal side of the second molar to accommodate the mesiodistal width of the crown of the third molar) to PG-III (the third molar is fully embedded in the mandibular ramus), there was observed a significant gradual decrease in retromolar space (RS) (p < 0.05). Specifically, in terms of mesiodistal angulation (angle between the line passing through the CEJ of the first and second molars and the line passing through the long axis of the third molar, RS exhibited a noticeable increasing trend with an increase in angulation [28]. This conclusion is in agreement with our study results denoted by the least MPAL in the mesioangular impaction group at the apex level. The significance of prioritizing apical dimensions lies in the conclusion that the retromolar space at the crown level exceeded that at the root level, with minimal presence observed at the root apex. This underscores the importance of giving particular attention to the initial RS at the apical level when undertaking molar distalization [29]. 

The limitations of the current study were, firstly, a gender-based evaluation was not carried out in this study. This was considered a limitation as the results of one of the previous studies by Vinay et al. stated that considerable differences existed between the sexes in terms of mandibular length, bigonial breadth, and bicondylar width, which may have an impact on MPAL distances [30]. Secondly, regarding the line of distalization that was considered in this study, since the implant for distalization mostly is placed on the buccal side, there is a tendency for buccal flaring. As a result of these conclusions, the MPAL may be still lesser in actual clinical situations than the findings of this study. To ensure reproducibility, the posterior occlusal line (POL) from earlier research was established as a reference line [3,17,20]. The other limitation of the study was that samples with absent third molars were not considered for evaluation in this study.

## Conclusions

The study yields significant insights into the dynamics of mandibular distalization, drawing three key conclusions. Firstly, it establishes that the available space for distalization diminishes as it approaches the root apex. Secondly, the MPAL distance varies among mandibles, with the highest measurements observed in those with fully erupted third molars and the mesioangular impaction group at a specific level, 8mm below the CEJ. Intriguingly, the MPAL distance is notably reduced in the mesioangular impaction group at the apex level.

In terms of clinical significance, the study posits that practitioners can predict an approximate 3mm of achievable distalization in the mandible. However, it emphasizes a limitation in distalization potential for mandibles with mesioangularly impacted third molars. These findings offer valuable insights for predicting MPAL distances in individuals with Class III malocclusion; offering an alternative means for evaluation in situations where the ethical justification for employing CBCT may be limited.

## References

[1] Papadopoulos MA (2014). Skeletal Anchorage in Orthodontic Treatment of Class II Malocclusion E-Book: Contemporary applications of orthodontic implants, miniscrew implants and mini plates.

[2] Gracco A, Lombardo L, Cozzani M, Siciliani G (2008). Quantitative cone-beam computed tomography evaluation of palatal bone thickness for orthodontic miniscrew placement. Am J Orthod Dentofacial Orthop.

[3] Kim SJ, Choi TH, Baik HS, Park YC, Lee KJ (2014). Mandibular posterior anatomic limit for molar distalization. Am J Orthod Dentofacial Orthop.

[4] Nakada T, Motoyoshi M, Horinuki E, Shimizu N (2016). Cone-beam computed tomography evaluation of the association of cortical plate proximity and apical root resorption after orthodontic treatment. J Oral Sci.

[5] Langer LJ, Pandis N, Mang de la Rosa MR, Jost-Brinkmann PG, Bartzela TN (2023). Eruption pattern of third molars in orthodontic patients treated with first permanent molar extraction: a longitudinal retrospective evaluation. J Clin Med.

[6] Begtrup A, Grønastøð HÁ, Christensen IJ, Kjær I (2013). Predicting lower third molar eruption on panoramic radiographs after cephalometric comparison of profile and panoramic radiographs. Eur J Orthod.

[7] Kim TW, Artun J, Behbehani F, Artese F (2003). Prevalence of third molar impaction in orthodontic patients treated nonextraction and with extraction of 4 premolars. Am J Orthod Dentofacial Orthop.

[8] Hashemipour MA, Tahmasbi-Arashlow M, Fahimi-Hanzaei F (2013). Incidence of impacted mandibular and maxillary third molars: a radiographic study in a Southeast Iran population. Medicina Oral, Patología Oral y Cirugía Bucal.

[9] Van der Weijden F, Dell'Acqua F, Slot DE (2009). Alveolar bone dimensional changes of post-extraction sockets in humans: a systematic review. J Clin Periodontol.

[10] Keser E, Naini FB (2022). Accelerated orthodontic tooth movement: surgical techniques and the regional acceleratory phenomenon. Maxillofac Plast Reconstr Surg.

[11] Kernitsky JR, Ohira T, Shosho D, Lim J, Bamashmous A, Dibart S (2021). Corticotomy depth and regional acceleratory phenomenon intensity. Angle Orthod.

[12] Perciaccante VJ (2007). Management of Impacted Teeth.

[13] MacGregor AJ (1985). The Impacted Lower Wisdom Tooth. https://books.google.co.in/books/about/The_Impacted_Lower_Wisdom_Tooth.html?id=3u9pAAAAMAAJ&redir_esc=y.

[14] (1993). Impacted Teeth. https://www.deepdyve.com/lp/oxford-university-press/impacted-teeth-1993-editors-c-c-ailing-j-f-helfrick-r-d-alling-QiZN8JEr5z.

[15] Santosh P (2015). Impacted mandibular third molars: review of literature and a proposal of a combined clinical and radiological classification. Ann Med Health Sci Res.

[16] (2017). Winter's classification. https://www.researchgate.net/figure/Winters-classification-Third-molars-are-classified-according-to-their-inclination-to_fig1_315733169.

[17] Choi YT, Kim YJ, Yang KS, Lee DY (2018). Bone availability for mandibular molar distalization in adults with mandibular prognathism. Angle Orthod.

[18] Karad A (2014). Clinical Orthodontics: Current Concepts, Goals and Mechanics. https://play.google.com/store/books/details?id=SjJTBwAAQBAJ&pli=1.

[19] Hui VL, Xie Y, Zhang K (2022). Anatomical limitations and factors influencing molar distalization. Angle Orthod.

[20] Kim SH, Cha KS, Lee JW, Lee SM (2021). Mandibular skeletal posterior anatomic limit for molar distalization in patients with Class III malocclusion with different vertical facial patterns. Korean J Orthod.

[21] Pillai AK, Thomas S, Paul G, Singh SK, Moghe S (2014). Incidence of impacted third molars: a radiographic study in People’s Hospital, Bhopal, India. J Oral Biol Craniofac Res.

[22] Wainwright WM (1973). Faciolingual tooth movement: its influence on the root and cortical plate. Am J Orthod.

[23] Lemos Rinaldi MR, Azeredo F, Martinelli de Lima E, Deon Rizzatto SM, Sameshima G, Macedo de Menezes L (2018). Cone-beam computed tomography evaluation of bone plate and root length after maxillary expansion using tooth-borne and tooth-tissue-borne banded expanders. Am J Orthod Dentofacial Orthop.

[24] Contemporary Orthodontics [Internet]. Google Books. [cited 2023 May 19]. Available from. https://books.google.com/books/about/Contemporary_Orthodontics.html.

[25] Jing Y, Han X, Guo Y, Li J, Bai D (2013). Nonsurgical correction of a Class III malocclusion in an adult by miniscrew-assisted mandibular dentition distalization. Am J Orthod Dentofacial Orthop.

[26] Poletti L, Silvera AA, Ghislanzoni LT (2013). Dentoalveolar class III treatment using retromolar miniscrew anchorage. Prog Orthod.

[27] Yu J, Park JH, Bayome M, Kim S, Kook YA, Kim Y, Kim CH (2016). Treatment effects of mandibular total arch distalization using a ramal plate. Korean J Orthod.

[28] Huang Y, Chen Y, Yang D (2023). Three-dimensional analysis of the relationship between mandibular retromolar space and positional traits of third molars in non-hyperdivergent adults. BMC Oral Health.

[29] Breik O, Grubor D (2008). The incidence of mandibular third molar impactions in different skeletal face types. Aust Dent J.

[30] G V, R MG, J A (2013). Sex determination of human mandible using metrical parameters. J Clin Diagn Res.

